# Soluble TREM2 is associated with death and cardiovascular events after acute ischemic stroke: an observational study from CATIS

**DOI:** 10.1186/s12974-022-02440-y

**Published:** 2022-04-12

**Authors:** Yaling Lu, Yu Zhao, Qi Zhang, Chongquan Fang, Anran Bao, Wenjing Dong, Yanbo Peng, Hao Peng, Zhong Ju, Jiang He, Yonghong Zhang, Tan Xu, Chongke Zhong

**Affiliations:** 1 Department of Epidemiology, School of Public Health and Jiangsu Key Laboratory of Preventive and Translational Medicine for Geriatric Diseases, Medical College of Soochow University, 199 Renai Road, Industrial Park District, Suzhou, 215123 Jiangsu China; 2 Department of Orthopaedics, Suzhou Science & Technology Town Hospital, Gusu School, Nanjing Medical University, Suzhou, China; 3 Department of Neurology, Affiliated Hospital of North China University of Science and Technology, Hebei, China; 4 Department of Neurology, Kerqin District First People’s Hospital of Tongliao City, Tongliao, Inner Mongolia China; 5 Department of Epidemiology, Tulane University School of Public Health and Tropical Medicine, New Orleans, LA USA; 6 Department of Medicine, Tulane University School of Medicine, New Orleans, LA USA

**Keywords:** sTREM2, Ischemic stroke, Prognosis, Cardiovascular events, Death

## Abstract

**Background:**

Soluble triggering receptor expressed on myeloid cells 2 (sTREM2), which reflects microglia activation, has been reported closely associated with neuronal injury and neuroinflammation. We aimed to prospectively investigate the associations between plasma sTREM2 and clinical outcomes in acute ischemic stroke (AIS) patients.

**Methods:**

Study participants were from the China Antihypertensive Trial in Acute Ischemic Stroke, plasma sTREM2 levels in the acute phase of AIS were measured in 3285 participants. The study outcomes were death, cardiovascular events and severe disability at 1 year after AIS. Cox proportional hazards models or logistic regression models were performed to examine the associations of plasma sTREM2 and clinical outcomes.

**Results:**

After 1-year follow-up, 288 participants (8.8%) experienced cardiovascular events or died. Multivariable-adjusted hazard ratios or odds ratios (95% confidence intervals) for the highest quartile of sTREM2 were 1.57 (1.11–2.21) for the composite outcome of death and cardiovascular events, 1.68 (1.09–2.60) for death, and 1.53 (1.08–2.18) for death or severe disability compared to the lowest quartile. Moreover, incorporation sTREM2 into traditional risk factors model significantly improved risk prediction of the composite outcome of death and cardiovascular events as evidenced by net reclassification index and integrated discrimination improvement (all *p* values < 0.05). There were joint effects of sTREM2 and galectin-3 on death and cardiovascular events. Participants with simultaneous elevation of sTREM2 and galectin-3 levels had the highest risk of the composite outcome of death and cardiovascular events.

**Conclusions:**

Elevated sTREM2 levels were independently associated with increased risks of death and cardiovascular events after AIS.

## Background

Microglia, principal immune cells of the brain, are the first cells to be activated after stroke and play an essential role in inflammatory microenvironment of stroke as they can either accelerate inflammation, enhance neurogenesis and attenuate neuronal apoptosis, which thereby could determine the stroke outcomes [1–3]. Triggering receptor expressed on myeloid cells 2 (TREM2) is mainly expressed on the surface of brain microglia and has been identified as an important regulator of microglial function [4]. The soluble form of TREM2 (sTREM2) released by proteolytic cleavage of the cell surface receptor has been reported as a potential biomarker for microglia activity and closely associated with neuronal injury [5, 6]. Both in vivo and in vitro studies suggested sTREM2 involved in neuroinflammatory responses [7]. The current role of sTREM2 is mainly focused on neurodegenerative diseases including Parkinson’s disease and Alzheimer’s Disease (AD) [8–12]. Recently, the Hisayama Study suggested that elevated serum sTREM2 levels were significantly associated with increased risks of the dementia and its subtypes (AD and vascular dementia) in a general elderly Japanese population [12]. Previous experimental evidence based on middle cerebral artery occlusion mice indicated that TREM2 has crucial effects on stroke immune response, suggesting TREM2 could be a promising immunomodulation target in ischemic stroke [4, 13–16]. However, population-based studies designed to comprehensively evaluate the prognostic significance of sTREM2 after ischemic stroke are scarce.

Stroke, in which ischemic stroke almost account for 70%, is the leading cause of chronic disability and death in China [17, 18], and the risk of major vascular events is high after first stroke [19]. Effective biomarkers which provide valuable information and enhance clinical care are clearly needed. To better understand and further extend current knowledge on the role of plasma sTREM2 in ischemic stroke pathology, we, therefore, aimed to prospectively explore the relationship between plasma sTREM2 and stroke outcomes, including death, cardiovascular events and disability, using data from the China Antihypertensive Trial in Acute Ischemic Stroke (CATIS). Furthermore, galectin-3, as one of the key molecules of regulating microglial activation, was recently found to stimulate TREM2 signaling and detrimentally regulated inflammatory response in the context of AD, which thereby was regarded as a novel endogenous TREM2 ligand [20]. Intriguingly, our prior research revealed that higher levels of galectin-3 in the acute phase of ischemic stroke were associated with greater risk of mortality and major disability within 3 months [21]. Thus, we also investigated the joint effects of sTREM2 and galectin-3 on clinical stroke outcomes.

## Methods

### Study participants

The study participants were recruited from the CATIS trial, the design details and main results have been described previously [22]. In brief, the CATIS trial was a multicenter, single-blind, blinded end-points randomized clinical trial that was conducted in 26 hospitals across China from August 2009 to May 2013. The inclusion criteria for CATIS trial were as follows: (1) age ≥ 22 years; (2) first-ever ischemic stroke diagnosed by neurologists using computed tomography or magnetic resonance imaging; (3) time from onset to admission within 48 h; (4) systolic blood pressure (BP) between 140 and 220 mmHg. The exclusion criteria were as follows: (1) BP ≥ 220/120 mmHg; (2) treated with intravenous thrombolytic therapy; (3) severe heart failure, acute myocardial infarction or unstable angina, atrial fibrillation, aortic dissection, cerebrovascular stenosis, resistant hypertension, or in a deep coma. Finally, 4071 patients with elevated BP were recruited in the CATIS trial. In the present study, after excluding participants who did not offer blood samples or whose plasma sTREM2 concentrations were failed to determine, a total of 3285 patients were finally included.

### Standard protocol approvals, registrations, and patient consents

The CATIS protocol was approved by the Institutional Review Boards or Ethical Committees at Soochow University in China and Tulane University in the US and participating hospitals, in compliance with the Declaration of Helsinki, and was registered at clinicaltrials.gov (NCT01840072). All study participants provided written informed consent.

### Data collection

Fasting blood samples were drawn within 24 h of patients’ hospital admission, and were separated at clinical laboratories of each participating hospitals and immediately frozen at −80 °C. sTREM2, galectin-3 and high-sensitivity C-reactive protein (hsCRP) were measured centrally at Soochow University using commercially available immunoassays (R&D Systems, Minneapolis, Minnesota). The intra-assay and inter-assay coefficients of variations for all biomarkers were below 3.6% and 6.9%, respectively. Laboratory technicians who performed measurements were blinded to baseline characteristics and clinical outcomes.

Baseline information about demographic characteristics, clinical features, medical history, and medication use history were obtained at admission using a standard questionnaire by trained interviewers. Serum lipids, plasma glucose, and other clinical laboratory measurements were performed on fresh blood samples at each participating hospital at admission. BP was measured at admission by trained nurses according to a standard protocol adapted from procedures recommended by the American Heart Association [23]. The stroke severity was evaluated by trained neurologists with National Institutes of Health Stroke Scale (NIHSS) at admission [24].

### Study outcomes

Patients were followed up in person at 1 year by trained neurologists unaware of baseline characteristics and treatment assignment. The primary study outcome was a combination of death and cardiovascular events (e.g., vascular deaths, nonfatal stroke, nonfatal myocardial infarction, hospitalized and treated angina, hospitalized and treated congestive heart failure, and hospitalized and treated peripheral arterial disease). The secondary outcomes included death, cardiovascular events, and severe disability (modified Rankin Scale [mRS] score 4–6). Score of the mRS was ranged from 0 to 6, with a score of 0 indicating no symptom and a score of 6 indicating death. Death certificates were obtained for deceased participants, and hospital data were abstracted for all cardiovascular events. The study outcomes assessment committee, unaware of treatment assignment, reviewed and confirmed subsequent outcomes based on the criteria established in the Antihypertensive and Lipid-Lowering Treatment to Prevent Heart Attack Trial.

### Statistical analysis

All study participants were classified according to quartiles of plasma sTREM2 levels. Continuous variables were described by means with SD or median with interquartile range depended on their distribution, and the generalized linear regression analysis was conducted to test for trend across the quartiles of plasma sTREM2 levels. Categorical variables were presented as frequency with percentile, and the Cochran–Armitage trend χ^2^ test was conducted to assess for trend across the quartiles.

The cumulative incidences of study outcomes across the sTREM2 quartiles were estimated on the basis of Kaplan–Meier curves and compared by log-rank tests. Cox proportional hazards regression models and logistic regression models were used to estimate the relationships between plasma sTREM2 and study outcomes, by calculating hazard ratios (HRs), odds ratios (ORs) and corresponding 95% confidence intervals (CIs). We conducted 2 different models: (1) model 1 was a univariable model; (2) model 2, a multivariable model, was adjusted for age, sex, current smoking, alcohol drinking, time from onset to randomization, admission NIHSS score, systolic BP, hsCRP, medical history (hypertension, hyperlipidemia, coronary heart disease and diabetes mellitus), medication use history (antihypertensive and lipid-lowering medications), family history of stroke, ischemic stroke subtype and randomized treatment. Tests for linear trends in HR/OR across the sTREM2 quartiles were conducted with the median within each quartile used as the predictor. The proportional hazards assumptions for the Cox model was tested using Schoenfeld residuals and found no violations. Stratified analyses were carried out to examine whether the associations of plasma sTREM2 with the primary study outcome differed according to age (< 60 or ≥ 60 years), sex, current smoking (yes or no), alcohol drinking (yes or no), admission NIHSS score (< 5 or ≥ 5), baseline hsCRP (< 1 or ≥ 1 mg/L), history of hypertension (yes or no) and receiving immediate BP reduction (yes or no). The interaction between sTREM2 and interested covariates on the primary outcome was tested by the likelihood ratio test of models with multiplicative interaction terms.

In addition, we test the performance of models with sTREM2 to predict the risk of death and cardiovascular events. First, continuous net reclassification index (NRI) and integrated discrimination improvement (IDI) were used to evaluate the incremental predictive value of sTREM2 beyond conventional risk factors. Second, likelihood ratio tests were performed to evaluate whether the global model fit improved after the addition of sTREM2 levels, and the Hosmer Lemeshow χ^2^ statistic were used to evaluate the calibration of models. We further examined the joint effects of sTREM2 and galectin-3 on the risk of study outcomes. Multiple imputation for missing covariate values was performed using the Markov chain Monte Carlo method. All *p* values were 2-tailed, and *p* values < 0.05 was considered to be statistically significant. Statistical analysis was conducted using SAS statistical software (version 9.4; SAS Institute, Cary, NC).

## Results

### Baseline characteristics

A total of 3285 patients (2098 men and 1187 women) were included in this study, and the median age was 62.0 years (interquartile range 55.0–70.0 years). The median plasma sTREM2 concentration was 401.27 pg/mL (interquartile range 247.45–651.60 pg/mL). Compared to patients with lower plasma sTREM2 levels, those with higher sTREM2 were more likely to be older and female; have higher admission NIHSS score, galectin-3, total cholesterol, low-density lipoprotein and high-density lipoprotein cholesterol; have higher prevalence of embolic stroke, history of coronary heart disease, history of antihypertensive and lipid-lowering medications use; have lower baseline diastolic BP; have lower prevalence of cigarette smoking, alcohol drinking and family history of stroke (Table 1).

**Table 1:** Characteristics of participants according to quartiles of plasma sTREM2

Characteristics ^*^	sTREM2, pg/mL				*p* trend
	< 247.45	247.45–401.27	401.27–651.60	≥ 651.60	
No. of patients	822	820	822	821	
Demographic features					
Age, y	57.0 (50.0–64.0)	60.0 (53.0–68.0)	62.0 (56.0–71.0)	68.0 (59.0–74.0)	< 0.001
Male sex, n (%)	613 (74.6)	551 (67.2)	491 (59.7)	443 (54.0)	< 0.001
Current cigarette smoking, n (%)	354 (43.1)	310 (37.8)	289 (35.2)	253 (30.8)	< 0.001
Current alcohol drinking, n (%)	302 (36.7)	288 (35.1)	227 (27.6)	192 (23.4)	< 0.001
Clinical features					
Time from onset to randomization, h	10.0 (5.00–24.0)	12.0 (5.0–24.0)	10.0 (4.0–24.0)	10.0 (4.0–24.0)	0.12
Admission systolic BP, mm Hg	161.3 (152.0–178.7)	163.3 (152.0–178.7)	161.3 (152.0–176.0)	162.0 (152.0–178.7)	0.63
Admission diastolic BP, mm Hg	98.7 (90.0–101.3)	98.7 (90.0–101.3)	98.7 (90.0–100.7)	94.7 (88.7–100.0)	< 0.001
Triglyceride, mmol/L	1.4 (1.0–2.0)	1.6 (1.0–2.3)	1.5 (1.0–2.3)	1.5 (1.0–2.2)	0.78
Total cholesterol, mmol/L	4.8 (4.2–5.5)	5.0 (4.3–5.7)	5.1 (4.3–5.7)	5.1 (4.4–5.8)	< 0.001
LDL-cholesterol, mmol/L	2.8 (2.2–3.4)	2.9 (2.3–3.5)	2.9 (2.3–3.5)	2.9 (2.3–3.6)	0.009
HDL-cholesterol, mmol/L	1.2 (1.0–1.5)	1.2 (1.0–1.5)	1.2 (1.0–1.5)	1.3 (1.0–1.6)	< 0.001
Fasting plasma glucose, mmol/L	5.8 (5.1–7.2)	5.8 (5.1–7.4)	5.8 (5.2–7.2)	5.8 (5.0–7.1)	0.68
Admission NIHSS score	4.0 (2.0–8.0)	4.0 (2.0–7.0)	4.0 (3.0–7.0)	5.0 (3.0–8.0)	0.01
High-sensitivity C-reactive protein, mg/L	1.7 (0.6–4.1)	1.8 (0.8–4.9)	1.9 (0.7–4.5)	2.3 (0.8–5.5)	0.38
Medical history, n (%)					
Hypertension	658 (80.0)	648 (79.0)	643 (78.2)	633 (77.1)	0.13
Hyperlipidemia	44 (5.4)	65 (7.9)	65 (7.9)	63 (7.7)	0.09
Diabetes mellitus	140 (17.0)	158 (19.3)	148 (18.0)	136 (16.6)	0.66
Coronary heart disease	58 ( 7.1)	66 ( 8.0)	97 (11.8)	117 (14.3)	< 0.001
Family history of stroke, n (%)	185 (22.5)	163 (19.9)	135 (16.4)	132 (16.1)	< 0.001
Medication use history, n (%)					
Antihypertensive medications	372 (45.3)	408 (49.8)	403 (49.0)	431 (52.5)	0.007
Lipid-lowering medications	17 (2.1)	29 (3.5)	34 (4.1)	32 (3.9)	0.03
Ischemic stroke subtype, n (%)					
Thrombotic	644 (78.4)	648 (79.0)	628 (76.4)	599 (73.0)	0.004
Embolic	18 (2.2)	38 (4.6)	35 (4.3)	65 (7.9)	< 0.001
Lacunar	160 (19.5)	134 (16.3)	159 (19.3)	157 (19.1)	0.75
Randomized treatment, n (%)	404 (49.1)	420 (51.2)	400 (48.7)	423 (51.5)	0.56
Galectin-3, ng/ml	7.5 (5.2–10.1)	8.5 (5.9–11.3)	8.6 (6.3–11.8)	9.8 (6.5–13.9)	< 0.001

*sTREM2* soluble triggering receptor expressed on myeloid cells 2, *BP* blood pressure, *LDL* low-density lipoprotein, *HDL* high-density lipoprotein, *NIHSS* NIH Stroke Scale

^*^Continuous variables are expressed as mean ± SD or median (interquartile range). Categorical variables are expressed as frequency (%)

### Plasma sTREM2 levels and clinical outcomes

**Fig. 1:**
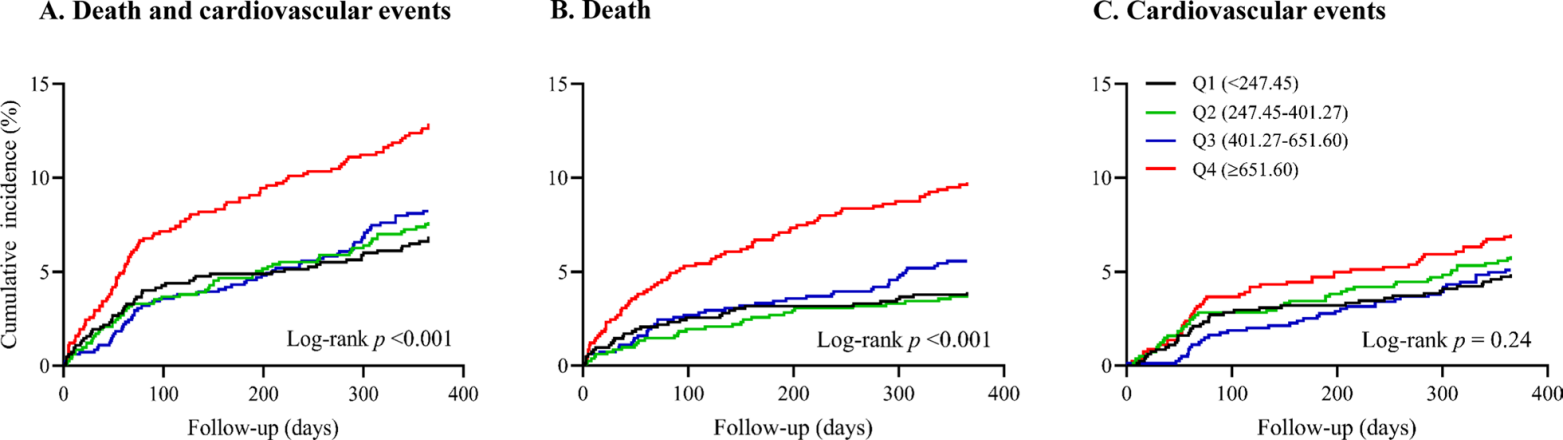
Kaplan–Meier survival curves. **A** Composite outcome of death and cardiovascular events, **B** Death, **C** Cardiovascular events. Q1: sTREM2 < 247.45 pg/mL; Q2: 247.45 ≤ sTREM2 < 401.27 pg/mL; Q3: 401.27 ≤ sTREM2 < 651.60 pg/mL; Q4: sTREM2 ≥ 651.60 pg/mL

After 1 year of follow-up, there were 288 participants (8.8%) experienced the composite outcome of cardiovascular events and death, including 186 (5.7%) deaths and 180 (5.5%) cardiovascular events. Kaplan–Meier curves showed that participants in the highest quartile of sTREM2 had significantly higher cumulative incidence rates of the composite outcome of death and cardiovascular events, and death (both log-rank *p* value < 0.001) (Fig. 1). Compared with the lowest quartile, the multivariable-adjusted HR or OR (95% CI) for the highest quartile of sTREM2 was 1.57 (1.11–2.21) for the primary outcome, 1.68 (1.09–2.60) for death, 1.53 (1.08–2.18) for the composite outcome of death and severe disability (Table 2).

**Table 2:** Risk of clinical outcomes according to quartiles of sTREM2 in the acute phase of ischemic stroke

	sTREM2, pg/mL				*p* trend
	< 247.45	247.45–401.27	401.27–651.60	≥ 651.60	
Median (pg/mL)	172.16	323.62	503.15	901.40	
The primary outcome: death or cardiovascular events					
No. of cases (%)	56 (6.8)	63 (7.7)	66 (8.0)	103 (12.6)	< 0.001
Unadjusted HR	1.00	1.13 (0.79–1.61)	1.19 (0.83–1.70)	1.93 (1.40–2.68)	< 0.001
Multiple-adjusted HR^*^	1.00	1.01 (0.70–1.46)	1.01 (0.70–1.45)	1.57 (1.11–2.21)	0.002
Death					
No. of cases (%)	32 (3.9)	31 (3.8)	45 (5.5)	78 (9.5)	< 0.001
Unadjusted HR	1.00	0.97 (0.59–1.59)	1.43 (0.91–2.24)	2.56 (1.69–3.86)	< 0.001
Multiple-adjusted HR ^*^	1.00	0.76 (0.46–1.25)	1.06 (0.67–1.69)	1.68 (1.09–2.60)	< 0.001
Cardiovascular events					
No. of cases (%)	39 (4.7)	47 (5.7)	40 (4.9)	54 (6.6)	0.19
Unadjusted HR	1.00	1.21 (0.79–1.85)	1.04 (0.67–1.61)	1.46 (0.97–2.21)	0.09
Multiple-adjusted HR ^*^	1.00	1.14 (0.74–1.75)	0.97 (0.62–1.52)	1.36 (0.88–2.10)	0.17
Death or severe disability					
No. of cases (%)	76 (9.4)	97 (12.1)	103 (13.0)	145 (18.6)	< 0.001
Unadjusted OR	1.00	1.32 (0.96–1.81)	1.43 (1.05–1.96)	2.19 (1.63–2.95)	< 0.001
Multiple-adjusted OR ^*^	1.00	1.14 (0.80–1.64)	1.09 (0.76–1.56)	1.53 (1.08–2.18)	0.01
Severe disability					
No. of cases (%)	44 (5.7)	66 (8.5)	58 (7.8)	67 (9.5)	0.01
Unadjusted OR	1.00	1.55 (1.04–2.30)	1.40 (0.93–2.09)	1.75 (1.18–2.59)	0.02
Multiple-adjusted OR*	1.00	1.39 (0.90–2.15)	1.10 (0.71–1.72)	1.40 (0.90–2.19)	0.29

*sTREM2* soluble triggering receptor expressed on myeloid cells 2, *HR* hazard ratio, *OR* odds ratio

^*^ Adjusted for age, sex, current smoking, alcohol drinking, time from onset to randomization, admission NIHSS score, systolic blood pressure, high-sensitivity C-reactive protein, history of hypertension, hyperlipidemia, coronary heart disease, and diabetes mellitus, use of antihypertensive and lipid-lowering medications, family history of stroke, stroke subtype and randomized treatment

**Fig. 2:**
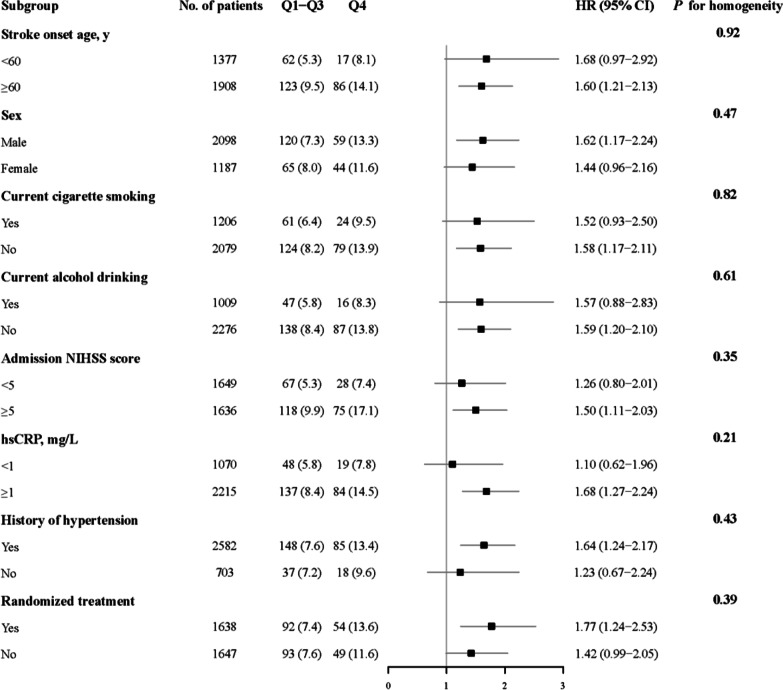
Subgroup analyses of the association between plasma sTREM2 and the primary outcome. The values below Q1–Q3 and Q4 were patients experiencing death or cardiovascular events, n (%). Hazards ratios (HRs) were calculated for higher plasma sTREM2 levels (≥ 651.60 pg/mL) after adjustment for the same variables as model 2 in Table 2, except for the stratified variable. CI indicates confidence interval; NIHSS indicates National Institutes of Health Stroke Scale; and hsCRP indicates high-sensitivity C-reactive protein

From these data, a threshold effect at the level of the highest quartile was observed. Thus, we created a binary variable (Q4 versus Q1–Q3) for the following subgroup analyses. We found that higher plasma sTREM2 levels were significantly associated with primary outcome in most strata of aforementioned covariates (age, sex, current smoking, alcohol drinking, admission NIHSS score, hsCRP, history of hypertension, and receiving immediate BP reduction). Furthermore, these covariates did not significantly modify the effect of sTREM2 on the risk of primary outcome (all *p* for interaction > 0.05; Fig. 2).

### Incremental prognostic value of sTREM2

We examined whether adding plasma sTREM2 to conventional risk factors improved the risk prediction for the composite outcome of death and cardiovascular events in patients with acute ischemic stroke (Table 3). Incorporation sTREM2 into the age and NIHSS score model significantly improved the risk stratification, as evidenced by NRI statistic (category-free NRI 19.4%, 95% CI 7.8% to 30.9%, *p* = 0.002) and IDI statistic (IDI 0.3%, 95% CI 0.1% to 0.6%, *p* = 0.009). Moreover, the likelihood ratio test showed that model fit was significantly improved, and the Hosmer Lemeshow test showed an adequate model calibration after the addition of sTREM2. Similarly, the significant improvement of predictive value was observed when adding sTREM2 to a basic model constructed with age, sex, NIHSS score, hsCRP, medical history, medication use history and other important prognostic factors (category-free NRI 19.2%, *p* = 0.002; IDI 0.4%, *p* = 0.006).

**Table 3:** Performance of models with sTREM2 ^*^ to predict the primary outcome in patients with acute ischemic stroke

	NRI (Category free)		IDI		Likelihood ratio test, *p* value	Calibration statistic	
	Estimate (95% CI), %	*p* value	Estimate (95% CI), %	*p* value		χ^2^	*p* value
Age + NIHSS score	Reference	–	Reference	–	Reference	23.30	0.003
Age + NIHSS score + sTREM2 ^*^	19.4 (7.8 to 30.9)	0.002	0.3 (0.1 to 0.6)	0.009	< 0.001	9.41	0.309
Basic model	Reference		Reference		Reference	9.29	0.318
Basic model + sTREM2 ^*^	19.2 (7.6 to 30.8)	0.002	0.4 (0.1 to 0.7)	0.006	< 0.001	6.28	0.616

*sTREM2* soluble triggering receptor expressed on myeloid cells 2, *NIHSS* National Institute of Health Stroke Scale, *NRI* net reclassification index, *IDI* integrated discrimination improvement

^*^ Quartile Basic model included age, sex, time from onset to randomization, current smoking, alcohol drinking, admission NIHSS score, systolic blood pressure, high-sensitivity C-reactive protein, medical history (hypertension, hyperlipidemia, coronary heart disease and diabetes mellitus), medication use history (antihypertensive and lipid-lowering medications), family history of stroke, ischemic stroke subtype and randomized treatment

### Joint effect of sTREM2 and galectin-3

Table 4 shows the joint effects of sTREM2 and galectin-3 on the study outcomes. Patients with both higher sTREM2 and galectin-3 levels had the highest incidence of all study outcomes, except severe disability. In the multivariable model, patients with higher levels of both sTREM2 and galectin-3 had a 2.23-fold risk of the composite outcome of death and cardiovascular events (HR 2.23; 95% CI 1.55–3.22) compared to patients with lower sTREM2 and galectin-3 levels. Similarly, the joint associations were also observed for death (HR 2.72; 95% CI 1.76–4.20), cardiovascular events (HR 2.05; 95% CI 1.27–3.30), and the composite outcome of death and severe disability (OR 1.62; 95% CI 1.09–2.41). There were no statistically significant interactions between sTREM2 and galectin-3 on study outcomes (all *p* for interaction > 0.05).

**Table 4:**
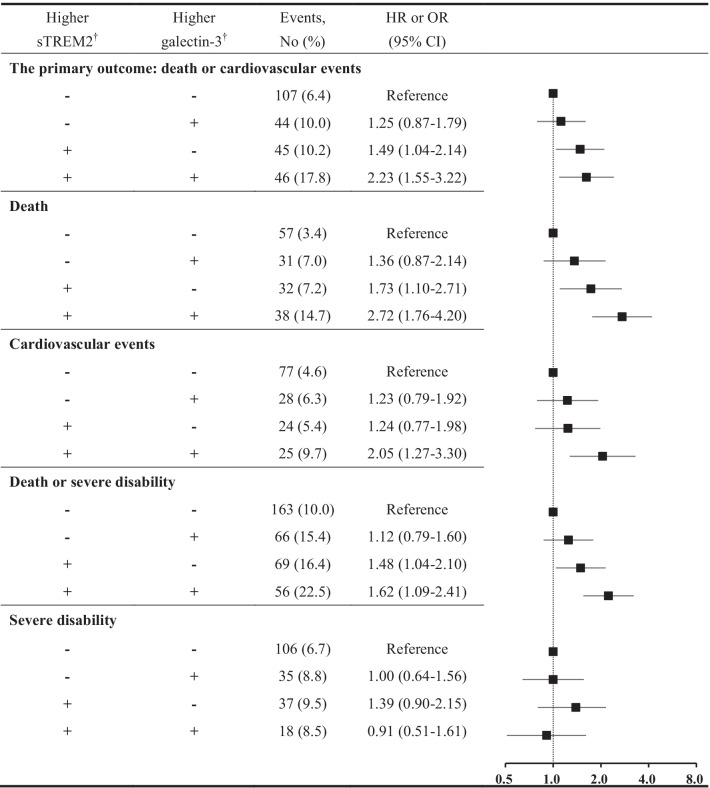
Joint effects of sTREM2 and galectin-3 on the risk of study outcomes after acute ischemic stroke

Analyses based on a sample restricted to individuals with both sTREM2 and galectin-3 values (*n* = 2808)

*sTREM2* soluble triggering receptor expressed on myeloid cells 2, *HR* hazard ratio, *OR* odds ratio, *CI* confidence interval

^†^sTREM2 and galectin-3 were classified into two categories (high versus low) based on the 75th percentile

HR or OR was adjusted for age, sex, current smoking, alcohol drinking, time from onset to randomization, admission NIHSS score, systolic blood pressure, high-sensitivity C-reactive protein, history of hypertension, hyperlipidemia, coronary heart disease, and diabetes mellitus, use of antihypertensive and lipid-lowering medications, family history of stroke, stroke subtype and randomized treatment

## Discussion

In this large, multicenter, prospective cohort of acute ischemic stroke patients, we demonstrated that higher sTREM2 levels were associated with greater risk of the composite outcome of death and cardiovascular events. The significant associations remained even after the adjustment for age, stroke severity, medical history, ischemic stroke subtype, inflammatory biomarker and several other potential confounding factors. In addition, after incorporating sTREM2 into the model with validated prognostic factors, including age and NIHSS score, the risk reclassification ability was significantly improved as suggested by NRI and IDI statistic. Moreover, we observed cumulative effects of sTREM2 and galectin-3 on stroke outcomes, patients with simultaneous elevations of sTREM2 and galectin-3 levels had substantially increased risk of death, cardiovascular events, as well as the composite outcome of death and severe disability. These findings indicated that plasma sTREM2 could be a potential predictor of clinical outcomes in patients with ischemic stroke.

Emerging population-based studies had found significant associations of sTREM2 and neurodegenerative diseases, such as Parkinson’s disease, AD and dementia [8–12]. Cross-sectional studies indicated that sTREM2 levels in the cerebrospinal fluid were significantly higher in the preclinical, mild cognitive impairment, and dementia stage of AD [10, 11]. Recently, the Hisayama Study reported that higher sTREM2 levels were significantly associated with increased risks of 10-year all-cause dementia, AD and vascular dementia in 1349 elderly Japanese, and suggesting circulating sTREM2 could be a possible biomarker for estimating the risk of dementia [12]. Interestingly, a gene-based study identified four genes, which included TREM2, shared by AD and ischemic stroke, implying TREM2-relevant immune signaling is one of the common pathogenesis underlying AD and ischemic stroke [25]. Given that sTREM2 is implicated in AD pathobiology and has been proposed as a candidate biomarker for AD, it is a reasonable hypothesis that circulating sTREM2 may provide novel insight in the prognostication of ischemic stroke.

Experimental evidence showed that TREM2 expression is remarkably upregulated and is involved in ischemic brain damage by modulating microglial phenotypes. For example, in peri-infarcted rat brains, TREM2 expression changes nearly fourfold compared to controls [25]. In addition, Sieber et al. also reported a greatly increased transcription of TREM2 in middle cerebral artery occlusion mouse stroke models. Moreover, an attenuated pro-inflammatory cytokines and chemokine expression, accompanied by decreased microglial activation were observed in TREM2-knockout mice in the sub-acute phase (7d reperfusion) after stroke, and they, therefore, suggested that TREM2 might sustain a distinct inflammatory response after stroke [16]. However, very limited population-based research on the association of sTREM2 and ischemic stroke has been conducted. Prior epidemiological studies indicated that circulating sTREM2 levels were significantly associated with several ischemic stroke risk factors, including insulin resistance, glycosylated hemoglobin A1c, and total cholesterol [26, 27]. Kwon et al. analyzed the data of 43 consecutive patients with non-cardioembolic ischemic stroke and found that early increment of plasma sTREM2 was more common in patients with poor outcome, and was associated with 3-month poor functional outcome (mRS score ≥ 3) [28]. Of note, this study had small sample size and was not fully adjusted for potential confounders, such as age, medical and medicine history, and ischemic stroke substyle. In contrast, the present prospective study was based on a relatively larger sample from the CATIS trial, which was characterized by standardized protocols and rigid quality control procedures for data collection and outcome assessment, enabling us to make a more convincing evaluation of plasma sTREM2 with comprehensive stroke outcomes. We found that higher plasma sTREM2 in acute ischemic stroke was significantly associated with poor stroke outcomes and offered additional prognostic significance beyond conventional risk factors.

Galectin-3 was involved in apoptosis, cell proliferation, macrophage chemotaxis, phagocytosis, and neutrophil extravasation, and galectin-3 inhibition decreased pro-inflammatory cytokine expression [29, 30]. In addition, plasma galectin-3 concentrations were increased in patients with carotid atherosclerosis, and were independently associated with increased risk of cardiovascular mortality in patients with peripheral artery disease [31]. Our previous study reported that elevated galectin-3 levels were associated with increased risk of death and major disability at 3 months after ischemic stroke onset [21]. Furthermore, single-cell transcriptomic analyses of microglia under different neurodegenerative conditions indicated that galectin-3 was one of the most upregulated genes in TREM2-dependent processes [32, 33]. Interestingly, we observed joint effects of sTREM2 and galectin-3 on the risk of study outcomes, with patients with both higher sTREM2 levels and higher galectin-3 levels having more than twofold risk of the death or cardiovascular events.

Our study provided epidemiological evidence supporting a potential role of sTREM2-relevant immune signaling in the progression of ischemic stroke. These findings extended our knowledge of ischemic stroke pathophysiologic mechanisms and refined risk prediction after ischemic stroke, and had important clinical implications.

Several biological mechanisms may be responsible for these observed relationships. First, sTREM2 in the cerebrospinal fluid had been reported as a biomarker of microglial activation, and closely correlated with markers of neuronal injury and tau pathology, including neurofilament light chain, total tau and phospho-tau_181P_ [5, 6]. Higher sTREM2 may reflect the neuroinflammation caused by microglial activation and further contribute to brain vascular injury. In addition, previous experimental studies suggested a neuroprotective role of TREM2 activation or up-regulation after cerebral ischemia/reperfusion injury [4, 13–15], while sTREM2 might inhibit the beneficial function of TREM2 through acting as a decoy receptor and competitively binding the ligands of TREM2 [7, 34]. Our findings about the joint effects of sTREM2 and galectin-3 (endogenous TREM2 ligand) on the death and cardiovascular events are consistent with this hypothesis. Furthermore, sTREM2 was also found to be involved in the inflammatory responses [7]. Population-based studies indicated that circulating sTREM2 levels were positively associated with inflammatory markers, such as hsCRP [26, 27]. It might be plausible that elevated sTREM2, which derived from adipose tissue and monocytes [35], increased the risk of stroke clinical outcomes through systemic inflammation. However, in the present study, these significant associations of sTREM2 and study outcomes remained after adjustment for hsCRP. Further experimental and human studies are needed to clarify the potential biological mechanisms underlying the sTREM2-stroke outcomes relationships.

However, some limitations needed to be interpreted. First, the present study was based on CATIS trial, in which excluded patients with BP ≥ 220/120 mmHg or treatment with intravenous thrombolytic therapy, a selection bias may be unavoidable. However, the proportion of patients with blood pressure ≥ 220/120 mmHg or with intravenous thrombolytic therapy is low in China. Second, prior study had reported the temporal dynamics of sTREM2 expression [28]. However, in the present study, plasma sTREM2 levels were only measured once in the acute phase of ischemic stroke, which limited us to examine its dynamic changes over time and the effect on clinical stroke outcomes. Future studies conducted to test the prognostic role of sTREM2 at different stages after stroke are warranted. Third, this study is observational, the residual confounding may still exist despite a multiple of covariates had been adjusted, and future clinical trials are necessary to validate whether the observed association is causal.

## Conclusions

In summary, higher plasma sTREM2 concentrations in the acute phase of ischemic stroke were associated with greater risk of death and cardiovascular events. Furthermore, elevations of both sTREM2 and galectin-3 contributed appreciably to death and cardiovascular events.

## Data Availability

The data sets used and/or analysed during the current study are available from the corresponding author on reasonable request.

## Abbreviations

sTREM2: Soluble triggering receptor expressed on myeloid cells 2
AD: Alzheimer’s disease
CATIS: China Antihypertensive Trial in Acute Ischemic Stroke
BP: Blood pressure
hsCRP: High-sensitivity C-reactive protein
NIHSS: National Institutes of Health Stroke Scale
mRS: Modified Rankin Scale
HR: Hazard ratio
OR: Odds ratio
CI: Confidence interval
NRI: Net reclassification index
IDI: Integrated discrimination improvement

## References

[1] Ma Y, Wang J, Wang Y, Yang GY (2017). The biphasic function of microglia in ischemic stroke. Prog Neurobiol.

[2] Kanazawa M, Ninomiya I, Hatakeyama M, Takahashi T, Shimohata T (2017). Microglia and monocytes/macrophages polarization reveal novel therapeutic mechanism against stroke. Int J Mol Sci.

[3] Qin C, Zhou LQ, Ma XT, Hu ZW, Yang S, Chen M (2019). Dual functions of microglia in ischemic stroke. Neurosci Bull.

[4] Gervois P, Lambrichts I (2019). The emerging role of triggering receptor expressed on myeloid cells 2 as a target for immunomodulation in ischemic stroke. Front Immunol.

[5] Suarez-Calvet M, Kleinberger G, Araque Caballero MA, Brendel M, Rominger A, Alcolea D (2016). sTREM2 cerebrospinal fluid levels are a potential biomarker for microglia activity in early-stage Alzheimer's disease and associate with neuronal injury markers. EMBO Mol Med.

[6] Gisslén M, Heslegrave A, Veleva E, Yilmaz A, Andersson LM, Hagberg L (2019). CSF concentrations of soluble TREM2 as a marker of microglial activation in HIV-1 infection. Neurology.

[7] Zhong L, Chen XF, Wang T, Wang Z, Liao C, Wang Z (2017). Soluble TREM2 induces inflammatory responses and enhances microglial survival. J Exp Med.

[8] Wilson EN, Swarovski MS, Linortner P, Shahid M, Zuckerman AJ, Wang Q (2020). Soluble TREM2 is elevated in Parkinson's disease subgroups with increased CSF tau. Brain.

[9] Tan YJ, Ng ASL, Vipin A, Lim JKW, Chander RJ, Ji F (2017). Higher Peripheral TREM2 mRNA Levels Relate to Cognitive Deficits and Hippocampal Atrophy in Alzheimer's Disease and Amnestic Mild Cognitive Impairment. J Alzheimers Dis.

[10] Heslegrave A, Heywood W, Paterson R, Magdalinou N, Svensson J, Johansson P (2016). Increased cerebrospinal fluid soluble TREM2 concentration in Alzheimer's disease. Mol Neurodegener.

[11] Nordengen K, Kirsebom BE, Henjum K, Selnes P, Gísladóttir B, Wettergreen M (2019). Glial activation and inflammation along the Alzheimer's disease continuum. J Neuroinflam.

[12] Ohara T, Hata J, Tanaka M, Honda T, Yamakage H, Yoshida D (2019). Serum soluble triggering receptor expressed on myeloid cells 2 as a biomarker for incident dementia: the Hisayama study. Ann Neurol.

[13] Kawabori M, Kacimi R, Kauppinen T, Calosing C, Kim JY, Hsieh CL (2015). Triggering receptor expressed on myeloid cells 2 (TREM2) deficiency attenuates phagocytic activities of microglia and exacerbates ischemic damage in experimental stroke. J Neurosci.

[14] Wu R, Li X, Xu P, Huang L, Cheng J, Huang X (2017). TREM2 protects against cerebral ischemia/reperfusion injury. Mol Brain.

[15] Zhai Q, Li F, Chen X, Jia J, Sun S, Zhou D (2017). Triggering receptor expressed on myeloid cells 2, a novel regulator of immunocyte phenotypes. Confers Neuroprot Reliev Neuroinflam Anesthesiol.

[16] Sieber MW, Jaenisch N, Brehm M, Guenther M, Linnartz-Gerlach B, Neumann H (2013). Attenuated inflammatory response in triggering receptor expressed on myeloid cells 2 (TREM2) knock-out mice following stroke. PLoS ONE.

[17] Li Z, Jiang Y, Li H, Xian Y, Wang Y (2019). China's response to the rising stroke burden. BMJ.

[18] Wang W, Jiang B, Sun H, Ru X, Sun D, Wang L (2017). Prevalence, incidence, and mortality of stroke in china: results from a nationwide population-based survey of 480 687 adults. Circulation.

[19] Chen Y, Wright N, Guo Y, Turnbull I, Kartsonaki C, Yang L (2020). Mortality and recurrent vascular events after first incident stroke: a 9-year community-based study of 0·5 million Chinese adults. Lancet Glob Health.

[20] Boza-Serrano A, Ruiz R, Sanchez-Varo R, Garcia-Revilla J, Yang Y, Jimenez-Ferrer I (2019). Galectin-3, a novel endogenous TREM2 ligand, detrimentally regulates inflammatory response in Alzheimer's disease. Acta Neuropathol.

[21] Wang A, Zhong C, Zhu Z, Xu T, Peng Y, Xu T (2018). Serum Galectin-3 and Poor outcomes among patients with acute ischemic stroke. Stroke.

[22] He J, Zhang Y, Xu T, Zhao Q, Wang D, Chen CS (2014). Effects of immediate blood pressure reduction on death and major disability in patients with acute ischemic stroke: the CATIS randomized clinical trial. JAMA.

[23] Pickering TG, Hall JE, Appel LJ, Falkner BE, Graves JW, Hill MN (2005). Recommendations for blood pressure measurement in humans: an AHA scientific statement from the Council on High Blood Pressure Research Professional and Public Education Subcommittee. J Clin Hypertens (Greenwich).

[24] Brott T, Adams HP, Olinger CP, Marler JR, Barsan WG, Biller J (1989). Measurements of acute cerebral infarction: a clinical examination scale. Stroke.

[25] Wei CJ, Cui P, Li H, Lang WJ, Liu GY, Ma XF (2019). Shared genes between Alzheimer's disease and ischemic stroke. CNS Neurosci Ther.

[26] Tanaka M, Honda T, Yamakage H, Hata J, Yoshida D, Hirakawa Y (2018). A potential novel pathological implication of serum soluble triggering receptor expressed on myeloid cell 2 in insulin resistance in a general Japanese population: The Hisayama study. Diabetes Res Clin Pract.

[27] Tanaka M, Yamakage H, Masuda S, Inoue T, Ohue-Kitano R, Araki R (2019). Serum soluble TREM2 is a potential novel biomarker of cognitive impairment in Japanese non-obese patients with diabetes. Diabetes Metab.

[28] Kwon HS, Lee EH, Park HH, Jin JH, Choi H, Lee KY (2020). Early increment of soluble triggering receptor expressed on myeloid cells 2 in plasma might be a predictor of poor outcome after ischemic stroke. J Clin Neurosci.

[29] Rubinstein N, Ilarregui JM, Toscano MA, Rabinovich GA (2004). The role of galectins in the initiation, amplification and resolution of the inflammatory response. Tissue Antigens.

[30] Dong H, Wang ZH, Zhang N, Liu SD, Zhao JJ, Liu SY (2017). Serum Galectin-3 level, not Galectin-1, is associated with the clinical feature and outcome in patients with acute ischemic stroke. Oncotarget.

[31] Madrigal-Matute J, Lindholt JS, Fernandez-Garcia CE, Benito-Martin A, Burillo E, Zalba G (2014). Galectin-3, a biomarker linking oxidative stress and inflammation with the clinical outcomes of patients with atherothrombosis. J Am Heart Assoc.

[32] Krasemann S, Madore C, Cialic R, Baufeld C, Calcagno N, El Fatimy R (2017). The TREM2-APOE pathway drives the transcriptional phenotype of dysfunctional microglia in neurodegenerative diseases. Immunity.

[33] Mathys H, Adaikkan C, Gao F, Young JZ, Manet E, Hemberg M (2017). Temporal tracking of microglia activation in neurodegeneration at single-cell resolution. Cell Rep.

[34] Piccio L, Buonsanti C, Cella M, Tassi I, Schmidt RE, Fenoglio C (2008). Identification of soluble TREM-2 in the cerebrospinal fluid and its association with multiple sclerosis and CNS inflammation. Brain.

[35] Park M, Yi JW, Kim EM, Yoon IJ, Lee EH, Lee HY (2015). Triggering receptor expressed on myeloid cells 2 (TREM2) promotes adipogenesis and diet-induced obesity. Diabetes.

